# Pain experiences of patients with musculoskeletal pain + central sensitization: A comparative Group Delphi Study

**DOI:** 10.1371/journal.pone.0182207

**Published:** 2017-08-10

**Authors:** Axel Georg Meender Schäfer, Leonie Johanna Joos, Katharina Roggemann, Kerstin Waldvogel-Röcker, Michael Pfingsten, Frank Petzke

**Affiliations:** 1 University of Applied Sciences Bremen, Faculty of Social Sciences, Degree Programme Applied Sciences Speech and Language Therapy and Physiotherapy, Bremen, Germany; 2 University of Applied Science Hildesheim, Faculty of Social Work and Health, Degree Programme for Occupational Therapy, Speech and Language Therapy and Physiotherapy, Hildesheim, Germany; 3 Pain Clinic, Department of Anaesthesiology, University Medicine Göttingen, Göttingen, Germany; Teesside University, UNITED KINGDOM

## Abstract

**Objectives:**

Central sensitization (CS) is regarded as an important contributing factor for chronification of musculoskeletal pain (MSP). It is crucial to identify CS, as targeted multimodal treatment may be indicated. The primary objective of this study was therefore to explore pain experience of individuals with MSP+CS in order to gain a better understanding of symptoms in relation to CS from a patient perspective. The secondary objective was to investigate whether pain experiences of patients with MSP+CS differ from those of individuals with neuropathic pain (NP).

**Methods:**

We conducted a comparative Group Delphi Study including patients with MSP+CS and neuropathic pain (NP). 13 guiding questions were used to gather information about sensory discriminatory, affective and associated bodily, mental and emotional phenomena related to the pain experience of patients. Descriptions were categorized using qualitative content analysis. Additionally, patients completed several pain related questionnaires.

**Results:**

Nine participants with MSP+CS and nine participants with NP participated. The Delphi procedure revealed three main themes: psycho-emotional factors, bodily factors and environmental factors. Descriptions of patients with MSP+CS showed a complex picture, psycho-emotional factors seem to have a considerable impact on pain provocation, aggravation and relief. Impairments associated with mental ability and psyche affected many aspects of daily life. In contrast, descriptions of patients with NP revealed a rather mechanistic and bodily oriented pain experience.

**Discussion:**

Patients with MSP+CS reported distinct features in relation to their pain that were not captured with current questionnaires. Insight in patient’s pain experience may help to choose and develop appropriate diagnostic instruments.

## Introduction

Musculoskeletal pain (MSP) such as low back, neck or shoulder pain is the most common reason for pain in general and contributes significantly to health related costs in western industrialized countries, second only to cardiovascular diseases [1]. While most patients recover within an expected period of time, in about 10% the condition becomes chronic [2]. Reasons for chronification are multidimensional, as many biopsychosocial mechanisms contribute to the condition in each individual patient. One important dimension of mechanisms are neurophysiological pain mechanisms, and classification of patients to pain mechanism based therapy has been promoted as one promising treatment approach for patients with chronic pain syndromes [3, 4]. Central sensitization is regarded as the most important pain mechanism contributing to chronification of musculoskeletal pain [5]. However, “central sensitization” is an umbrella term comprising a multitude of different mechanisms taking place in the dorsal horn of the spinal cord, ascending and descending pathways in the dorsal column, the brainstem and pain centres in the forebrain [6], all leading ultimately to amplification of innocuous and painful stimuli and to the extension of receptive fields [7]. For a number of reasons, it seems crucial to identify CS in patients with musculoskeletal pain. Firstly, it offers a plausible physiologic rationale to explain signs and symptoms and resulting disability in absence of relevant and explanatory pathological findings. Secondly, the presence of CS should have practical implications with regard to medical and therapeutic interventions. Patients with CS possibly should avoid further nociceptive input from pain provoking aggressive interventions or too vigorous physical activities [8]. These could potentially aggravate the problem, as more input could lead to further augmentation of the pain system. Instead, interdisciplinary interventions such as graded activity exposure, cognitive behavioural treatment or pharmacological treatment have been recommended allowing tailored and gradual activation and possibly "desensitisation" [9].

So far, no gold standard exists to diagnose CS. Even for elaborated but costly and time-consuming procedures such as quantitative sensory testing or laser-evoked potentials there are no generally agreed and diagnostic cut-off values. Thus, clinicians commonly rely on signs, symptoms as well as clinical examination to identify CS [8]. In a recent Delphi survey [10], pain experts identified the following key clinical features: “Disproportionate, non-mechanical, unpredictable pattern of pain provocation in response to multiple/non-specific aggravating/easing factors”, “pain persisting beyond expected tissue healing/pathology recovery times”, “Pain disproportionate to the nature and extent of injury or pathology”and wide spread pain. Cardinal clinical signs include allodynia, hyperpathia and hyperalgesia.

The above mentioned symptoms and signs are based mainly on the perspectives of clinicians and/or researchers and contain multiple subjective components based on clinical judgement. In regards to the broad impact of CS in the genesis and maintenance of chronic musculoskeletal pain, it seems important to gain more detailed information about pain experience from the perspective of individuals with MSP+CS. This information may be valuable to determine patient preferences and values and to help guide the use or development of questionnaires assessing the domain of central sensitisation.

The primary objective of this study was therefore to explore pain experience and understanding of individuals with MSP+CS. The secondary objective was to investigate whether pain experiences of patients with MSP+CS differ from those of individuals with neuropathic pain (NP).

## 1 Materials and methods

### 1.1 Research team

LJ, KW, KR and SN conducted the Delphi rounds. The interviewers were all female and trained physiotherapists (BSc), with a median experience of 8 (IQR 4.5) years in the treatment of musculoskeletal or neurological patients. The interviewers and AS were not known to the participants until the start of the study, FP as head of the pain clinic was known to the participants from consultation and treatment, but was not involved in the data collection process. The conduction of the study was supervised by AS and FP.

### 1.2 Study design

A qualitative cross-sectional study with two groups (Group MSP+CS: Patients with musculoskeletal pain + central sensitization; Group NP: Patients with neuropathic pain) was conducted. Data was collected by adapting the Delphi-technique for groups. The Delphi-technique for groups is an explorative approach to determine unknown information and represents a discursive process to gather information about a topic, which is relevant to the participants of the study [11]. The aim of the process is to find consensus within a group of experts [12]. In this study, patients were regarded as experts for their individual pain experience.

### 1.3 Ethical approval

Ethical approval for this study was obtained from the Human Research Ethics Committee, Universitätsmedizin Göttingen (Nr. 18/9/11) in accordance with the Declaration of Helsinki on Ethical Principles for Medical Research Involving Human Subjects. All subjects provided written informed consent to participate.

### 1.4 Sample

A convenience sample of nine patients with musculoskeletal pain and central sensitization and nine patients with neuropathic pain were recruited from active patients in a multidisciplinary and tertiary pain clinic. All patients considered had undergone thorough clinical and psychological diagnostics at admission and were treated for at least six months at the institution. The diagnostic psychological interview was conducted by a trained psychologist (MP) specialized in treating patients with chronic pain, the clinical assessment by a pain specialist MD. Patients had received different treatments ranging from purely medication interventions to participation in the pain clinic’s multimodal treatment program (five in group MSP+CS and two in NP). Active patients were reviewed by MP and FP and approached if likely to fulfil the inclusion criteria for either group. They were then re-evaluated by FP and MP and recruited, if in-criteria were fulfilled and no exclusion criteria present. Since the aim was to recruit two groups of “representative” patients in terms of the selected criteria and not a representative overall sample no detail on excluded patients were collected (see limitations of the study in the Discussion section). Participants had to be between 18 and 80 years of age and either have a diagnosed musculoskeletal (MSP + CS) disorder or neuropathic pain disorder (NP). Specific exclusion and inclusion criteria for the group MSP+CS are shown in Table 1.

**Table 1:** Exclusion and inclusion criteria for MSP+CS.

1. Exclusion criteria
□ Presence of neuropathic pain following the clinical algorithm described by Treede et al. [13] □ Evidence of neural tissue disorders irrespective of the presence/absence of neuropathic pain
2. Inclusion criteria
2a. Symptoms
□ “Disproportionate pain in relation to the nature and extent of the injury or pathology”[14] □ Multiple, non-anatomic areas of pain [14] □ “Pain persisting beyond expected tissue healing/pathology recovery times”[10] □ “Pain of high severity and irritability (i.e. easily provoked, taking a long time to settle)”[10]
2b. Signs
□ “Diffuse/non-anatomic areas of pain/tenderness on palpation”[14] □ “Disproportionate, non-mechanical, unpredictable pattern of pain provocation in response to multiple/non-specific aggravating/easing factors”[14]

Inclusion criteria for the group Neuropathic Pain were:

- Age between 18 and 80
- Definite neuropathic pain according to [13]

Exclusion criteria for both groups were the clinical diagnosis of a somatoform pain disorder based on the initial diagnostic psychological interview on pain clinic admission and re-evaluation. The psychological interviews were conducted by a trained psychologist specialized in treating patients with chronic pain (MP).

### 1.5 Data collection

#### 1.5.1 Delphi procedure

Central to the Delphi-technique are questions that are used to maintain the focus of the discussion on pain experience and to ensure consistency across groups. To generate questions, a systematic literature search was conducted, searching the databases Medline, Cinahl, Cochrane, PEDro, Sport Discus and the following journals: Pain, physioscience, Manuelle Therapie, Spine and Rheumatologie. Search terms were:

(myofascial pain syndromes [MeSH] OR “musculoskeletal pain”) AND ("central sensitization" OR "sensory hypersensitivity" OR allodynia [MeSH] OR pain threshold [MeSH] OR chronic disease [MeSH] OR chronic pain OR "widespread pain")

43 relevant articles were screened for pain dimensions and descriptions. The most frequent ones were discussed within the research team to reach consensus. A list of key questions was derived to cover the identified dimensions and descriptions with 13 questions concerning sensory discriminatory (Q 1–12) and associated bodily, mental, emotional and activity related phenomena (Q 13a-d) of central sensitization (Table 2). The list of questions was pretested with a sample of six chronic pain patients and adapted to enhance comprehensiveness and acceptance.

**Table 2:** List of questions, method of data collection and data format.

Q#	Construct	Question (German original)	Question (English translation)	Method of data collection	Data format
	Pain localisation	Bitte zeichnen Sie auf der beigefügten Körperskizze Ihre Schmerzen nach den Vorgaben ein.	Please mark your pain areas in the body chart.	pain drawing	drawing
Q1	Pain intensity	Wie stark ist Ihr Schmerz auf einer Skala von 0 bis 10 wobei 0 kein Schmerz und 10 der stärkster vorstellbare Schmerz bedeutet?	How intense is your pain on a scale from 0 to 10 where 0 is no pain and 10 is most intense pain imaginable?		
Q1a	Mean (SD) pain intensity last 7 days (NRS)	Durchschnittlicher Schmerz in den letzten sieben Tagen?	Mean pain during the last 7 days?	numerical rating scale	numeric 0–10
Q1b	Most intense pain last 7 days (NRS)	Stärkster Schmerz in den letzten sieben Tagen?	Most intense pain during the last 7 days?	numerical rating scale	numeric 0–10
Q1c	Least pain last 7 days (NRS)	Geringster Schmerz in den letzten sieben Tagen?	Least pain during the last 7 days?	numerical rating scale	numeric 0–10
Q2	Frequency of pain	Wie oft tritt der Schmerz auf?	How often does the pain occur?	narrative	descriptors
Q3	Pain duration	Wie lange hält der Schmerz an?	How long does the pain last?	narrative	descriptors
Q4	Course of a pain episode	Wie verhält sich die Schmerzintensität während einer Schmerzphase?	Does the pain intensity change during one pain episode?	narrative	drawing
Q5	Time of pain occurrence	Zu welchem Zeitpunkt tritt der Schmerz auf?	At which point in time does the pain occur?	narrative	descriptors
Q6	Pain trigger	Was löst den Schmerz aus?	What triggers the pain?	narrative	descriptors + rating
Q7	Pain relieve	Was lindert den Schmerz?	What relieves the pain?	narrative	descriptors + rating
Q8	Pain exacerbation	Was verstärkt den Schmerz?	What exacerbates the pain?	narrative	descriptors + rating
Q9	Change of pain	Wie hat sich ihr Schmerz seit Beginn der Problematik verändert?	How did your pain change since the beginning of your problem?	narrative	descriptors + rating
Q10	Allodynia	Welche Aktivitäten, Reize oder Bewegungen verursachen Schmerzen, die früher keine ausgelöst haben?	Are there activities, stimuli or movements that cause pain that have formerly not caused pain?	narrative	descriptors + rating
Q11	Hyperalgesia	Auf welche schmerzauslösenden Reize reagieren sie heute empfindlicher als früher?	Are there any painful stimuli which are now more painful than before?	narrative	descriptors + rating
Q12	Pain characteristic	Mit welchen Eigenschaften würden sie ihren typischen Schmerz beschreiben, wenn sie an ein Schmerzereignis denken?	What are the typical characteristics to describe a pain incident?	narrative	descriptors + rating
Q13	Associated phenomena:	Welche weiteren Begleiterscheinungen und Besonderheiten beobachten Sie seit Beginn der Schmerzproblematik bei sich bezüglich:	Which associated phenomena do you observe in relation to:		
Q13a	Body	ihres Körpers?	Your body?	narrative	descriptors + rating
Q13b	Mental ability	ihrer geistigen Leistungsfähigkeit?	Your mental ability?	narrative	descriptors + rating
Q13c	Psyche	ihrer Psyche?	Your psyche?	narrative	descriptors + rating
Q13d	Activities of daily living	ihren Alltagsaktivitäten?	Your activities of daily living?	narrative	descriptors + rating

The Delphi procedure took place during a one-day session at a multimodal pain clinic from 9am 5pm, with appropriate breaks between the data collection sessions. Patients with MSP+CS and patients with NP participated separately on two different occasions. To the knowledge of the investigators there was no communication or interaction between patients of the two groups. The research team had no information on the clinical diagnosis of the participating patients beyond their overall group assignment.

Data collection followed a structured and standardized procedure [11]. There were two groups with nine patients each, one group consisted of patients with musculoskeletal pain and central sensitization, the other group of patients with neuropathic pain. The procedures were the same for each group. In each group, the nine patients were randomly split into three groups with three participants and one researcher per group to facilitate and moderate the discussion and to take notes of participants statements. The list of questions was explained to the patients. To give the participants an idea about the quality and expected content of the discussion and to facilitate the participants discussion a fictitious case example was read to them [11]. Within this case example, the day of a 52-year old woman with musculoskeletal pain and central sensitisation is described. She exhibits typical features of central sensitization such as hyperalgesia and widespread pain, its impact on social activities (visiting friends) and cognitive function (unable to concentrate at work) are described.

Data was collected in two rounds. During the first round (90 Minutes) the questions were discussed. The aim was to collect as many subjective pain-describing items from individual participants as possible. Besides, the participants were asked to draw the location and expansion of their pain into a body chart and to report quantitative aspects of their pain experience such as pain intensity or duration of pain (Table 2).

For the second round of data collection participants were allocated randomly to new groups. Patients were asked to rate the various items from the first round by using a Likert-Scale from 0 to 10 (0 = no relevance; 10 = very high relevance) [11]. After the second round of data collection, the ratings from the three groups were collected and a new item list with group ratings was printed and presented to the whole group.

Finally, all nine participants could ask questions, make comments and were informed about the further procedure. They received the final item list including the ratings to take home. Participants were asked to reflect on the results and make changes or comments if applicable and to post the commented list back to the research team.

#### 1.5.2 Questionnaires

To gain further information, patients additionally completed the following questionnaires on another occasion. This data collection was scheduled a few weeks after the survey to not influence the patient’s subjective performance by providing a range of predefined possible answers.

- Based on a body diagram the area marked as painful was scored from 0–19 following the regions described in the regional pain scale [15]
- Pain Sensitivity Questionnaire (PSQ) [16] measures pain sensitivity by self rating;
- Tampa Scale of Kinesiophobia [17] a questionnaire for kinesiophobia;
- Pain Perception Scale (SES) [18] measures affective and sensory pain perception;
- Depression Anxiety Stress Scales (DASS) [19] screens for depression and anxiety;
- Patient Health Questionnaire (PHQ) [20] evaluates severity of somatic symptoms;
- Pain Detect Questionnaire [21] screens for neuropathic pain components
- Pain Catastrophizing Scale (PCS) [22] a questionnaire to assess catastrophizing in patients with chronic pain
- SF-36 [23] measures physical and mental components of quality of life

### 1.6 Data analysis

Descriptions of the course of pain were visualised in seven different graphs. Quantitative data (Q1a-c) from round one was summarized by calculating the median and interquartile range (IQR) as well as arithmetic mean and standard deviation (SD).

Means were also calculated for the collective ratings of the qualitative data (Q4-13) from round two. These mean ratings were used to reduce the number of items; all items with a mean rating below 3/10 as well as duplicates were removed. To analyse pain descriptions, a qualitative content analysis according to Mayring [24] was conducted. Single items were inductively assigned to main- and subcategories. Hierarchical category trees were then constructed to further categorize the data and to visualize the results.

Questionnaire data was compared between groups using non parametric statistics (Mann-Whitney-U-Test), since n was too small to assess normal distribution [25]. For the SES and SF-36 group data was also expressed as mean and SD to allow comparison to normative data typically presented that way.

## 2 Results

### 2.1 Participants

Data collection took place during two Delphi sessions. In the first session nine patients with MSP+CS participated, consisting of eight females and one male who had the following diagnoses: fibromyalgia (n = 2), spinal pain (n = 3), chronic widespread pain (n = 4). Patients with spinal pain had non-specific pain progressing beyond the initial low back or neck pain (but not radicular pain), patients with chronic widespread pain had no other specific diagnosis, except for mild and non-specific degenerative changes. Median age was 49 years (IQR = 6).

In the second session nine patients (five females and four males) with NP participated, median age was 57 (IQR = 19). Patients in the group NP had the following diagnoses: neuralgia (n = 4), polyneuropathy (n = 4) and radiculopathy (n = 1). For details see Table 3. Patients with neuralgia had specific and anatomically sound distributions of pain (face V1, cervical postzosteric neuralgia, distal tibial nerve, plantar nerve), polyneuropathy pain was restricted to the distal limbs in variable degrees.

**Table 3:** Age, sex and diagnoses of included patients.

	Musculoskeletal Pain + CS (n = 9)	Neuropathic Pain (n = 9)
Age: Median (IQR) mean±SD	49 (6) * 49.44 ± 3.36*	57 (19) * 58 ±14.87
Sex: % female (n)	89% (8)	56% (5)
Diagnoses (n)	Fibromyalgia (2)	Trigeminal neuralgia (1)
	LBP s/p Spondylodesis L4-S1 (2)	Radiculopathy L5 (1)
	Chronic widespread pain (4)	Diabetic small fibre polyneuropathy (1)
	Extended spinal pain mostly thoracic spine & sacroiliac joint (1)	Small fibre polyneuropathy s/p chemotherapy for breast cancer (1)
		Diabetic polyneuropathy (1)
		Neuralgia tibial nerve left lower leg (1)
		Postzosteric neuralgia C2 & C3 right (1)
		s/p Guillain Barre Syndrome and PNP both feet (1)
		Neuralgia plantar right s/p peripheral nerve injury (1)

IQR Interquartile Range; s/p status post

* U-Test: significant difference between groups p = 0.042

### 2.2 Questionnaires

The results of the completed questionnaires are summarized in Table 4. Patients with neuropathic pain reported significantly fewer areas as painful compared to MPS+CS. Patients in both groups showed similar PSQ scores indicative of increased pain hypersensitivity with total scores above 4/10 for 5 of 8 patients in the NP pain group and 5 of 7 patients in the MSP+CS group [26]. Likewise, values for the PSQ minor score were not statistically different between the two groups, yet slightly lower in the NP group. Fear of movement was less marked in both groups, patients mean scores were lower than typical data for mean Tampa scores in patients with musculoskeletal pain in other studies ranging from 39.4–42 [17].

**Table 4:** Questionnaires.

	Musculoskeletal Pain + CS (n = 8)					Neuropathic Pain (n = 7)					Mann-Whitney U Test
	Median	P 25	P 75	Mean	SD	Median	P 25	P 75	Mean	SD	p
Regional Pain Score	13.5	6.25	17.25	12.12	5.67	2	1	4	2.86	2.27	0.004
Pain Sensitivity Questionnaire PSQ (0–10) Total Score	5.04	3.5	6.89	5.14	1.68	4.21	2.43	6.43	4.72	2.15	0.69
Minor Score	3.8	2.6	5.71	4.0	1.68	1.71	1.14	6.14	3.24	2.84	0.23
Tampa Scale of Kinesiophobia	14	8.5	23	15.38	8.31	30	17	35	26.57	8.89	0.054
Schmerzempfindungsskala											
SES sensorisch	28	26	37	31.50	7.71	33	29	49	37.43-	10.83	0.23
SES affektiv	19	15.5	27	21.63	8.16	25	19	29	23.86	7.31	0.54
Depression Anxiety Stress Scales DASS											
Depression	5.5	2	10	6.50	5.66	9	5	16	9.29	6.16	0.42
n ≥ 10				n = 2 25%					n = 2 29%		
Anxiety	4.5	2	11	6.12	5.36	5	3	9	6.00	3.83	0.86
n ≥ 6				n = 4 50%					n = 3 43%		
Stress	8.5	3.5	14	9.25	6.41	10	7	18	11.00	5.83	0.64
n ≥ 7				n = 6 75%					n = 6 86%		
Patient Health Questionnaire PHQ											
Somatisation Score (0–30)	8	6	11	8.38	2.77	12	9	15	11.43	4.2	0.14
Number of Items with Somatisation Score = 2	2.5	1.5	3	2.38	1.51	3	3	6	4.00	2.31	0.12
Number of patients with ≥ 3 Items with Somatisation Score = 2				n = 4 (44%)					n = 6 (67%)		
Pain Detect Questionnaire (0–38)											
Overall score	19	13.5	21.5	17.13	6.9	24	19	30	23.57	8.02	0.93
unclear 13–18 (n)				4					1		
neuropathic >18 (n)				4					6		
Pain Catastrophising Scale	20	13.5	27.5	19.63	9.43	22	19	37	27.00	13	0.27
SF-36 (0–100)											
Physical functioning	57.5	32.5	67.5	51.25	20.31	45	20	85	48.57	27.04	0.82
Role-physical	50	12.5	62.5	43.75	34.72	0	0	75	28.57	41.9	0.37
Bodily pain	41	22	41	33.63	13.88	32	22	41	31.29	16.55	0.76
General Health	44.5	33.5	54.5	44.63	12.47	32	30	45	34.57	10.06	0.13
Vitality	45	32.5	62.5	46.88	21.7	55	20	60	41.43	20.96	0.82
Social Functioning	62.5	56.25	68.75	65.63	16.02	62.5	25	87.5	57.14	32.96	0.72
Role-emotional	83.33	0	100	58.33	49.6	100	33.33	100	71.43	40.5	0.61
Mental health	72	50	82	68.00	19.83	52	44	76	59.43	15.04	0.38
Physical component summary (PCS)	32.43	29.46	37.89	32.93	6.31	28.73	23.83	35.32	29.54	8.6	0.34
Mental component summary (MCS)	54.03	36.29	55.77	48.03	11.2	46.44	43.34	58.38	46.81	10.69	0.87

P25/75: 25%/75% Percentile; SD Standard Deviation

Scores for subjective sensory and affective pain sensation measured with the pain sensation scale (SES) [18] were lower than typical pain population scores in both groups. The SES measures the emotional burden of pain (SES affective) as well as sensory qualities of pain related to pressure, rhythm or temperature (SES sensory), lower scores reflect less burden and sensory qualities. Patients in group MSP+CS had mean scores that were 1.85 SD (SES sensory) and 2.84 SD (SES affective) below the mean of typical pain population scores, patients in group NP were 1.26 SD (SES sensory) and 2.61 SD (SES affective) below.

Mean Depression subscale (DASS) was below the cut off score in both groups [27], however the proportion of patients exceeding cut off scores for the subscales anxiety and stress was substantial ranging from 43% (n = 3) to 86% (n = 6) within the groups (Table 4). Screening with the Patient Health Questionnaire (PHQ-15) revealed low somatic symptom severity in group MSP+CS (median = 8, IQR = 5) and medium somatic symptom severity in group NP (median = 12, IQR = 6) [20]. The proportion of patients with high somatic symptom severity was 0% (n = 0) in group MSP+CS and 29% (n = 2) in group NP.

Six patients of seven in the NP group had Pain Detect (PD) scores >18 indicating a likely neuropathic pain component, compared to four out of eight in the MSP+CS group. For the rest of the patients a neuropathic pain component was uncertain with PD scores between 12 and 18. For none of the participants a neuropathic pain component could be excluded (PD score <12) [21].

Patients in both groups exhibited impaired general physical health with SF36 physical component summary 1.71 SD (MSP+CS) and 2.05 SD (NP) below the norm [23]. Mental health status was comparable to population normative data with 0.2 SD (MSP+CS) and 0.3 SD below the norm.

### 2.3 Delphi procedure

The results of the Delphi procedure are summarized in Table 5.

**Table 5:** Summary of results Delphi procedure.

Q#	Question	Category	Subcategory	MSP+CS		NP	
				Mean (SD) Median (IQR)	No. of items	Mean (SD) Median (IQR)	No. of items
Q1a	Mean pain intensity last 7 days (NRS 0–10)			5.83 (1.41) 5 (2)		6.44 (2.07) 7 (2)	
Q1b	Most intense pain last 7 days (NRS 0–10)			7.67 (1.22) 7 (1)		7.94 (1.7) 8 (2)	
Q1c	Least pain last 7 days (NRS)			3.78 (1.43) 3.5 (1)		4.33 (2.24) 4 (2)	
				Mean	No. of items	Mean	No. of items
Q2	Frequency of pain	constant		X		X	
		intermittent		X		X	
Q3	Pain duration	constant		X		X	
		variable		X		X	
Q4	Course of a pain episode	constant with flashes		-		7.3	-
		constant with waves		5.83		-	
Q5	Time of pain occurence	any time		X		X	
		no pattern		X		X	
Q6	Pain trigger	psychoemotional		6.8	14	4.3	1
		bodily factors		6.4		4.9	
			motor stimuli	8.1	5	4.9	7
			sensory stimuli	4.6	2	4.9	2
		context factors		7.1	3	-	-
		no trigger		5.7	1	4.3	1
Q7	Pain relieve	bodily		7.2	16	5.3	3
		psychoemotional		6.4	27	3.8	4
Q8	Pain excacerbation	bodily		6.7	9	4.9	2
		psychoemotional		6.5	30	3.1	2
		context factors		6.9	2	3.9	7
		no factors		-	-	4.0	1
Q9	Change of pain	worsening		X	17	X	7
		improvement		X	8	X	8
		change		-	-	X	3
Q10	Allodynia	Stimuli		7.5	1	5.2	4
		Movement		7.2	20	4.9	9
		Activities		6.4	7	3.3	1
Q11	Hyperalgesia	mechanical stimuli		7.7	1	3.3	1
Q12	Pain characteristic						
		Emotional		7.63	3	8	3
		quality	Burning	6.9	1	6.3	1
			Shooting	6.75	2	6.73	3
			Dull	6.55	3	6.0	1
			Stiff	6.13	4	3.3	1
			Numb	6.1	3	-	-
			Cramping	5.93	3	-	-
			Sharp	5.84	5	5.26	5
			Throbbing	5.36	5	3.7	1
			tingling	-	-	5.7	1
		Spatial	Radiating	7.7	1	-	-
			Moving	6.6	1	-	-
Q13	Associated phenomena:						
Q13a	Body	Somatic		6.2	17	5.6	9
			head	8.3	1	-	-
			skin	6.8	3	-	-
			nasopharangyal	5.8	4	-	-
			cardiovascular	5.8	2	-	-
			eyes	5.7	6	-	-
			gastrointestinal	5.5	2	-	-
			Musculoskeletal	-	-	4.9	8
		Psychosomatic		6.4	5	5.2	8
		Sensory		7.0	10	4.9	3
			Temperature	7.3	5	-	-
			Visual	7.2	1	-	-
			Acoustic	7.0	1	-	-
			Paraesthesia	6.4	3	8	1
Q13b	Mental ability	Reduced motivation		7.2	8	6.0	3
			Inner drive	7.7	4	6.7	2
			Perseverance	6.7	4	5.3	1
		Reduced Vigilance					
			fatigue	7.5	2	5.1	2
			lack of concentration	6.8	3	6.4	4
			memory deficits	6.3	1	5.7	2
			ability to react	5.5	2	-	-
			attention	5.3 (deficit)	2	3.4 (improvement)	2
		Impaired ability to communicate		5.8		4.8	
			sending information	6.2	4	4.8	3
			receiving information	5.3	1	-	-
		Reduced resilience		7.2	4	-	-
			increased sensitivity	7.5	2	-	-
			Reduced patience	6.9	2	-	-
Q13c	Psyche	Emotions		5.5		3.8	
			Stability	6.0	5	-	-
			Mood	5.7	5	3.8	4
			Self esteem	4.5	2	-	-
		Volition (“Antrieb”)		6.0	4	3.0	1
		Social aspects		5.5		-	-
			Behaviour	6.5	2	-	-
			Interaction	4.4	8	-	-
		Attitude		-	-	4.6	11
Q13d	ADL	Time management		7.2	3	-	-
		Areas of life		5.6		5.3	
			Work / household	6.5	7	6.8	2
			Leisure time	5.8	6	5.9	17
			Hobbies	5.6	4	-	-
			Sport	4.6	2	4.5	2
		Social behaviour		5.4	4	4.3	3

X = Not applicable

#### 2.3.1 Pain localization

Patients in the MSP+CS group reported painful areas all over the body. In the body charts they marked eight to twenty painful areas, which often had a widespread extent. Patients in the NP group marked one to three pain regions in the body charts. These reflected the underlying neuropathic disorder.

#### 2.3.2 Pain intensity, frequency, duration, course and occurrence (Q1-5)

The median highest experienced pain in the last week on an 11-point NRS was 7 (IQR 1) for the MSP+CS group and 8 (IQR 2) for the NP group. The lowest experienced pain of the last week was 3.5 (IQR 1) for group MSP+CS and 4 (IQR 2) for group NP. The patients had median pain intensity during the last week of 5 (IQR 2) in the MSP+CS group and 7 (IQR 2) in the NP group. Participants in both groups typically described constant pain (i.e. “always” or “365 days a year” MSP+CS and NP). Answers concerning pain duration also showed that both NP and MSP patients experience constant pain.

While the intensity of pain seemed to be similar (Mann Whitney U Test p>0.26) and both groups reported a constant component in their pain, the course over time differed between the two groups. Whereas the NP group typically reported constant pain with peaks like “flashes” or “electric shocks”, the MSP+CS group described the pain as a constant pain with waves that seemed to last longer than the peaks of the NP group. This was in line with the use of items that indicate differing variability of pain: “interval” (NP) or “depending on the situation” (MSP+CS), “repeated bouts of 10–30 seconds duration” (NP) or “a few seconds up to four hours” (MSP+CS).

#### 2.3.3 Quality of pain (Q 12)

In both groups participants described characteristics of their typical pain. Two main categories could be derived: pain quality and affect.

For patients in group NP the most relevant subcategories for pain quality were shooting 6.73 (i.e. “fulgurant pain” 7.5), burning (6.3) and dull (6.0). Most relevant descriptions in the category affect (8.0) for patients with NP were “nasty” (9.7) and “nerving” (9.0).

Pain descriptions in group MSP+CS were more numerous (33 vs. 17) and could be categorized in more subcategories (11 vs. 8) compared to patients with NP. Most relevant subcategories for patients with MSP+CS were radiating (7.7), burning (6.9) and shooting (6.75). The category affect (7.63) included pain descriptions such as “tormenting” (8.4).

#### 2.3.4 Pain trigger, reduction and aggravation (Q 6–8)

To investigate patient’s pain experience in regards to influencing factors, three questions were asked: “what triggers the pain?” “what relieves the pain?” and “what aggravates the pain?” (Table 2).

Patients with NP reported pain triggers /Q6) that could be categorized as bodily factors (4.9). Subcategories were motor stimuli (4.9) such as “physical load” (5.5) and sensory stimuli (4.9) such as “unsuitable shoes” (5.0). In group MSP+CS psycho-emotional trigger were most important (6.8), patients mentioned intrinsic factors such as “to put oneself under pressure” (8.2) and extrinsic factors like “lack of time” (8.2) or “excessive demands” (8.2). Patients in the MSP+CS group mentioned a total of 14 different items in the category psycho-emotional pain trigger compared to one item in group NP.

In group NP the participants mentioned “medication” (9.3) as the most important pain relieving factor (Q7). Patients in the MSP+CS group stated that physical therapy (8.2), physical stimuli such as “bathtub” (9.5) or “hot springs” (9.7) and relaxation such as “relaxing music” (8.0) were most relevant, also “medication” (6.2) and motor activity i.e. “aqua-fitness” (6.2) were mentioned. Overall, passive activities such as “bathtub” or “hot springs” received the highest rating in regards to pain relieve in group MSP+CS. In contrast, patients in the NP group also expressed active strategies such as “doing joyful things” (4.7) or “change of activity” (3.7) to reduce pain.

The category psycho-emotional factors was more highly rated (6.4) in the MSP+CS group compared to the NP group (3.8). This observation was further emphasized when comparing the total number of items mentioned: 27 items for psycho-emotional factors in group MSP+CS and 3 items in group NP.

In regards to factors that aggravate pain (Q8), experiences differed between the two groups. For patients in group NP the most relevant category was bodily factors such as physical strain (i.e. “standing for a long time” (5.7). Also relevant in the NP group was the statement “no pain aggravating factors” (4.0). Furthermore, context factors (3.7) were mentioned such as “cold” (3.3). Psycho-emotional factors were less relevant for patients with NP with a mean rating of 3.1.

In comparison to group NP, Patients in the MSP+CS group mentioned a greater number of possible factors that might aggravate pain (45 vs. 10 total items). Psycho-emotional factors (6.5) were the most relevant pain aggravating factors for the MSP+CS group with high mean ratings and a large number of items. Examples for descriptions in this category are “emotional distress” (8.0) or “to take things to much to the heart” (7.2). Patients also described personal factors (6.8) such as “working more than one has to” (8.2) and environmental factors (6.1) (i.e. “excessive demands” 7.8) and context factors (6.9) such as “cold” (9.0) or “the job” (4.8).

#### 2.3.5 Change of pain since the problem started (Q9)

In answer to the question “how did your pain change since the beginning of your problem?” patients in both groups reported improvement as well as worsening. Improvement based on endogenous factors such as “I have learned to deal with my pain (through therapy)” was reported. Worsening of pain was described in terms of a spread of pain without explainable cause in both groups.

#### 2.3.6 Allodynia (Q 10)

Patients in both groups (NP and MSP+ CS) reported signs of allodynia in relation to stimuli, movement and activities. Mean ratings for descriptions related to allodynia were higher in group MSP+CS compared to group NP. Perception of normally non painful stimuli as painful (MSP+CS 7.5; NP 5.2) such as punctual touch (MSP+CS 7.5) or pressure and strain (NP 6.0) were reported. A second category in both groups was movement related allodynia (MSP+CS: 7.5; NP: 4.9) associated with posture, movement and movement under load.

#### 2.3.7 Hyperalgesia (Q 11)

Participants in group NP did not describe any signs of hyperalgesia. Patients with MSP+CS mentioned that “to stub against something” (7.7) was more painful than before the start of the problem. Patients in both groups stated that they are less sensitive to painful stimuli compared to the beginning of their problem.

#### 2.3.8 Associated phenomena (Q13a-d)

Questions about associated phenomena in relation to the body, mental ability, psyche and activities of daily living were asked to gain insight into patient’s experiences on how pain affected different dimensions of function, activities and participation. Patients were asked to report associated phenomena that developed from the beginning of the disease.

Associated phenomena related to the body (Q13a) were categorized as sensory phenomena or as symptoms in body systems. These categories differed in relevance between the two groups. For patients in the group MSP+CS the most important pain descriptions were related to sensory phenomena (7.0) such as temperature (7.3) (i.e. “being cold” 9.7), vision (7.2) (i.e. “hypersensitivity to light” 7.2) or hearing (7.0) (“hypersensitivity to noise” 7.0). Other descriptions related to body systems (6.3) such as the skin (“dry skin” 8.3) or nasopharyngeal zone (i.e. “dry tongue” 6.7).

Patients in the group NP reported descriptions mainly in the category body systems (mean 5.4); these were related to the musculoskeletal system (i.e. “to adopt a relieving posture during walking” 5.7).

Associated phenomena in regards to mental ability (Q13b) had equal relevance for the patients. Both groups reported lack of motivation (MSP+CS 7.2; NP 6.0), with decreased perseverance (MSP+CS 7.7; NP 6.7) and lack of inner drive (MSP+CS 6.7; NP 5.3). Patients experienced a decrease in vigilance (MSP+CS 6.2; NP mean 5.2) as a result of fatigue, lack of concentration and memory deficits. Additionally, both groups reported an impaired ability to communicate (MSP+CS 5.8; NP 4.8) affecting both sending (“I can’t remember certain words” MSP+CS 6.7) and receiving information (“I have difficulties listening” MSP+CS 5.3).

Patients in both groups described different phenomena associated to psychological aspects (Q13c). Patients in group MSP+CS provided more descriptions compared to patients in group NP. These were predominantly negative changes in relation to thoughts (6.3) (i.e. “not being able to stop brooding” 6.7), lack of motivation (6.0) (i.e. “this is the bottom line” 6.8) and emotions (5.5) (“being more sensitive” 7.3).

Patients in group NP also reported positive changes in regards to their attitudes (4.6) and motivation (3.0) such as “priorities have changed” (6.7) or “I have so much energy that I can manage anything” (3.0).

Patients in both groups reported impairments in their activities of daily living (Q13d; MSP+CS 5.6; NP 5.3) such as employment or housework (MSP+CS 6.5; NP 6.8) (i.e. “I had to slow down” MSP+CS 7.7 or “I can’t work as many hours as I would like to” NP 7.3), hobbies (MSP+CS 5.4) and sport activities (MSP+CS 4.6; NP 4.5). Also impairments in regards to social activities with a mean ranking of 5.4 in group of MSP+CS (i.e. “when I am together with friends, I want to go home earlier” 7.3) and 4.3 in group NP (i.e. “I am more cautious to accept invitations” 5.0) were mentioned.

## 3 Discussion

### 3.1 Pain experience of patients with MSP+CS

Patients with MSP+CS experience constant moderate to severe pain in many body regions. Descriptions of these patients reveal a complex picture of pain manifesting in many dimensions, particularly in regards to emotional and psychological aspects. Exemplary are descriptions of pain trigger, pain aggravating and relieving factors that reveal the importance of psycho-emotional aspects.

Pain trigger and pain aggravating factors in group MSP+CS were often external factors such as perceived strenuous work situations. These are factors that cannot be easily influenced by the patients. Consequently, statements in regards to pain reduction in group MSP+CS typically reveal rather passive pain coping strategies.

Patients with MSP+CS are affected in many aspects of daily life. In regards to relevant phenomena associated with pain, patients reported impairments in regards to their mental ability, such as reduced motivation, vigilance and resilience as well as impaired ability to communicate. These findings are comparable to the findings of others studies. Turk et al. [28] reported that patients with chronic pain including patients with chronic musculoskeletal pain experience limitations in regards to enjoyment of life, emotional well-being, fatigue, weakness and sleep related problems, also cognitive deficits have been shown [29].

### 3.2 Differences in pain experience between patients with MSP+CS and patients with NP

Our data show similarities and contrasts of pain perception between patients with MSP+CS and NP. Patients in both groups were severely impaired by chronic pain and both report marked restrictions in their activities of daily living. The quantitative aspects of the pain experience such as intensity, frequency, duration, and time of occurrence were comparable between groups.

Contrasts could be shown in relation to sensory and affective aspects of pain. In general, structural and somatic factors in relation to pain triggers, reduction, aggravation and associated phenomena were more relevant for patients in group NP, whereas for patients with MSP+CS affective and emotional factors were most relevant. Associated phenomena related to the body were mainly confined to the musculoskeletal system in group NP, whereas patients with MSP+CS reported symptoms in multiple body systems.

This pattern also becomes evident in regards to pain triggers. Patients with NP mentioned mainly pain triggers related to the body. These were generally modifiable by behavioural changes such as avoiding physical strain or changing unsuitable shoes. Patients mainly employed active strategies to reduce pain (“doing joyful things”). This implies higher coping abilities or acceptance of pain related disabilities in the group NP. Patients in group MSP+CS weighted psycho-emotional factors higher when describing pain trigger, reduction and aggravation. This depicts contrasts in pain experience and in the understanding of the nature of pain. Patients with NP showed a rather mechanistic and structural pain understanding that may be enhanced by a more localised and “explainable” pain experience, whereas patients with MSP+CS experienced pain as complex and multifactorial, with a higher weight on psycho-emotional factors. This is further supported by the higher mean number of pain descriptions in all categories in group MSP+CS with 5.3 compared to 3.7 in group NP.

### 3.3 Comparison of Delphi and questionnaire data

Interestingly, these trends to describe pain were poorly reflected in the actual questionnaire data from the two groups, where patients with neuropathic pain reported more affective disturbances. This may indicate that the relationship between affective symptoms and pain perception could differ between patient groups. On the other hand, the pain detect questionnaire and the extent of pain was in line with Delphi results.

When comparing questionnaire data with Delphi results, further differences and discrepancies become obvious. In contrast to Delphi results all SF-36 subscales except bodily pain and PCS were within 1 SD of normative population scores. For example, the SF-36 subscales vitality (-0.32 SD) and mental health status score (MCS) (-0.2 SD) were not different to population normative data, although Delphi results clearly showed that patients felt affected in these dimensions. Also in contrast to Delphi findings, SES scores indicated that the emotional burden of pain was 2.84 SD below the norm for pain populations. When comparing Delphi results to questionnaire data limitations of the used questionnaires to capture the complex picture of patients pain experience and understanding become evident. These differing results may also reflect potential positive effects of therapy in the current patient sample that had received various pain management interventions. Thus Delphi results may represent an overall concept of pain and disability these patients have, while questionnaires may reflect the individual’s current status of health and pain.

Importantly, patients with MSP+CS had a more extensive spatial extent of pain, which was possibly biased by selection in this sample. However, it has recently been shown that even in a low back pain cohort, many patients report increased spatial extent of pain, when specifically asked for other pain sites [30]. In another set of studies fibromyalgia like characteristics (including spatial extent) were associated with poorer postoperative outcome following various types of surgery [31]. Recently developed criteria for central sensitization likewise require more generalized pain [7]. This indicates, that although the criteria chosen to select patients with CS in this study were based on [10, 31] and validated in a sample of chronic low back pain [14], they seem appropriate for other patient groups as well.

The Delphi procedure thus has the potential to capture the overall characteristics of the MSP+CS and NP condition in general, irrespective of the current situation of the patients. It stated the relevance of symptom and experiences from the patient’s perspective and not the individual level of symptom intensity as measured by the respective questionnaires. Viewed from this perspective typical clinical perceptions of the two different pain conditions are reproduced and confirmed by the Delphi procedure. On the other hand it becomes obvious how similar the overall burden of pain is for the two groups, which probably is better highlighted by the individual questionnaire data.

### Neuropathic pain versus central sensitization

As expected, more patients in the NP group were classified as “probable neuropathic” based on their painDetect scores (Table 4), however patients in both groups exhibited signs and symptoms indicative for neuropathic pain as no patient was categorized as “unlikely neuropathic pain”. Patients with MSP+CS did not show a neuroanatomically plausible pain distribution and were only included when they failed the algorithm suggested by Treede at al. [13] and when there was no clinical evidence (based on history, examination and available results of diagnotic tests) for a relevant neurological lesion or disease [32]. Thus patients with MSP+CS displayed some of the pain characteristics seen in neuropathic pain, while not having a clinical diagnosis of neuropathic pain.

This discrepancy might be related to properties of the painDetect questionaire. The validation describes selection of items based on the literature and not a patient survey [21]. The initial cohort consisted of two patient groups. One included patients with “classic” neuropathic pain conditions, namely postherpetic neuralgia, painful polyneuropathy, nerve trauma, and low back pain with neuropathic origin (presumably radicular pain), while the second group had pain of nociceptive origin, like visceral pain, osteoarthritis, inflammatory arthropathies and mechanical low back pain. Patients with assumed mixed origin, including fibromyalgia and ankylosing spondylitis were excluded. Two specialists had to concur in the diagnosis. Based on these clinically selected groups a score of ≤12 indicated that a neuropathic component is unlikely, a score ≥19 indicates likely neuropathic pain, between the score is uncertain. Sensitivity was reported to be 84% and specificity 84%. When the questionaire was used for patients with fibromyalgia, high frequencies of a positive pain detect score were also seen, which the authors associated with neuropathic pain characteristics indicative of central sensitisation [33, 34]. In an independent validation in a general chronic pain sample, 400 patients with any type of chronic pain diagnosis were included [35], and 37% of all patients displayed distinct neuropathic characteristics, including patients with musculoskeletal pain. A high level of depression, pain chronicity, and reported pain intensity explained most of the variance in painDetect scores. The authors conclude that any type of chronic pain may have neuropathic characteristics and that the painDetect screening tool was not able to differentiate typical neuropathic entities from other pain syndromes with neuropathic features. The results of our study support this conclusion. This indicates that clinically defined neuropathic pain and central sensitization may share certain pain characteristics, but differ in other clinical features.

### Strengths and weaknesses

The Delphi procedure for groups is a valid method for investigating experiences of experts in relation to a particular problem [11]. Experts usually have an overview of many different aspects of a problem, e.g. a clinician with longstanding experience will have seen many patients with a particular health problem. This is not the case for patients, who are only able to relate to their individual experience. Patients are experts for their individual pain, but not for pain in general. As a consequence, the generalizability of the results is limited and should be replicated in a larger sample of patients.

A second issue relates to the clinical diagnosis of “central sensitization”. An experienced medical pain specialist (FP) made the clinical diagnosis based on predefined criteria. Although the diagnostic criteria for central sensitization (Table 1) showed reliability [32] and discriminative validity [14], construct validity may be limited as so far no gold standard exists to diagnose CS and a potential systematic bias in selection cannot be ruled out.

Further limitations relate to the sample. Firstly, the two groups were not comparable in regards to sex and age. Patients in group MSP+CS were nine years younger on average and had a higher proportion of females (Table 3), which may have influenced contrasts in regards to pain experience between the two groups. Furthermore, the selected sample had varying degrees of ongoing treatment for their pain condition when they had entered the study. Groups differed in regards to treatment intensity within the pain clinic. Patients with MSP+CS had on average more multimodal treatment units compared to patients with NP. This might explain differences such as lower kinesiophobia and the generally normal mental component in the SF-36 in group MSP+CS. Patients still had a high level of chronification, yet generalizability to untreated or less chronic patients is limited.

Finally, although the sample size of nine per group is within the recommended size for Delphi groups, potential bias like an overrepresentation of patients with widespread pain / fibromyalgia, or a non-representative sample for neuropathic pain in this study (no central pain) limits the generalizability of the findings to a chronic pain population.

### Conclusion

The results of the present study illuminate features of a subgroup of patients with musculoskeletal disorders. Firstly, the general impression is, that patients with MSP+CS have a more complex and multifactorial pain experience and understanding, in comparison to a group of patients with NP. Secondly, for patients in group MSP+CS, the affective pain component is most relevant, and pain seems to compromise many different aspects of life and health. Furthermore, patients with MSP+CS are affected by associated phenomena relating to psychological and mental impairments, particularly concentration, vigilance, motivation and communication. These findings are in accordance with other studies [28, 36, 37] and are reflected in a recently developed Central Sensitization Inventory [38]. Patients with MSP+CS seem to present with distinct features that need to be considered in diagnosis and targeted treatment. However, the ability of our current instruments to differentiate between different patient groups (in this study MSP+CS and NP) seems limited.
